# Accumulation of plasmacytoid dendritic cell is associated with a treatment response to DNA-damaging treatment and favorable prognosis in lung adenocarcinoma

**DOI:** 10.3389/fimmu.2023.1154881

**Published:** 2023-06-26

**Authors:** Yoon Jin Cha, Eun Young Kim, Yong Jun Choi, Chi Young Kim, Min Kyung Park, Yoon Soo Chang

**Affiliations:** ^1^ Department of Pathology, Yonsei University College of Medicine, Seoul, Republic of Korea; ^2^ Department of Internal Medicine, Yonsei University College of Medicine, Seoul, Republic of Korea

**Keywords:** immune checkpoint inhibitors (ICIs), lung adenocarcinoma, plasmacytoid dendritic cells (pDCs), single cell RNA sequencing(scRNA-seq), smoking, toll-like receptor 9 (TLR9), tumor microenvironment(TME)

## Abstract

**Introduction:**

Favorable responses to the treatment including immune checkpoint inhibitors (ICIs) have been consistently reported in lung cancer with smoking history. As the tumor microenvironment (TME) may be involved in the treatment response to ICIs, we aimed to investigate the TME of lung cancer with different smoking status.

**Methods:**

Lung adenocarcinoma (LUAD) tissue (Tu) and adjacent normal-appearing lung tissue (NL) from current and never smokers were investigated by single-cell RNA sequencing and immunofluorescence and immunohistochemical staining. The clinical implications of identified biomarkers were validated using open-source datasets.

**Results:**

The lungs of smokers had an increased proportion of innate immune cells in NL tissues, whereas Tu tissues had a lower proportion of these cells than those of non-smokers. Monocyte-derived macrophages (mono-Mc), CD163-LGMN macrophages, monocyte-derived dendritic cells (DCs), and plasmacytoid DCs (pDCs) were significantly enriched in smokers’ Tu. Among these clusters, pDCs, specifically enriched in the Tu of smokers. The expression of representative pDC markers, leukocyte immunoglobulin-like receptor A4 (LILRA4) and Toll-like receptor 9 (TLR9), was increased in the stromal cells of LUAD in patients with a smoking history. In an animal model of lung cancer, ionizing radiation induced robust TLR9 expressing immune cells in peritumoral area. Survival analysis using a TCGA-LUAD dataset indicated that patients overexpressing pDC markers exhibited superior clinical outcomes to age-, sex-, and smoking-matched control groups. Top 25% patients with high TLR9 expression exhibited significantly higher tumor mutational burden than that of low TLR9 expression group (bottom 25% patients) (5.81 mutations/Mb vs 4.36 mutations/Mb; *P* = 0.0059, Welch’s two-sample *t*-test).

**Conclusion:**

There is an increased pDC in the TME of smokers’ lung cancer, and the response of pDC to DNA damaging treatment would lead a conducive environment to ICIs containing regimens. These findings suggest that R&D that induces an increase in the activated pDC population is continuously required to enhance therapeutic effectiveness of ICIs-containing therapies in lung cancer.

## 1 Introduction

In the past decade, immune checkpoint inhibitors (ICIs) have become the most widely used treatment agents for lung cancer owing to their superior anti-cancer effects. Single-agent PD-1 or PD-L1 inhibitors or a combination of chemotherapy and PD-1/PD-L1 inhibitors are preferred treatment options for patients with locally advanced and metastatic non-small cell lung cancer (NSCLC) without identifiable driver mutations (1). Evaluation of the response to ICI involves investigating PD-L1 expression (2–4), tumor mutational burden (TMB) (5), microsatellite instability, DNA mismatch repair, the composition of the intestinal microbiome (6), and prior antibiotic usage (7). However, data are still limited, and treatment responses to ICI-including regimens are difficult to predict as few patients benefit from ICIs. Additionally, there is an increasing economic burden and ICI-related adverse effects on ICI non-responders (8). Hence, the discovery of biomarkers and the development of therapeutic strategies are required to improve the clinical outcomes of ICI non-responders.

Therapeutic biomarkers can be identified by thoroughly examining drug-specific responder subpopulations among patients with cancer (9, 10). Smokers with lung adenocarcinoma (LUAD) consistently exhibit a favorable response to ICIs compared to non-smokers. A meta-analysis of ICI efficacy in different advanced cancers (11), including advanced NSCLC (12, 13), found that smokers had greater ICI benefits compared to non-smokers. This suggests that smoking-induced changes in the tumor microenvironment (TME) may influence ICI potency. These findings suggest that smoking-induced differences in the TME may provide clues for predictive biomarkers for ICIs or new therapeutic targets.

First, LUAD presents a significantly higher TMB in smokers than in non-smokers. Second, LUAD in smokers has a distinct mutational signature owing to exposure to tobacco, single-base substitution signature 4, which is characterized by G–T transversion (14, 15). Third, the proportional distribution of major driver mutations differs according to smoking status. According to a report from China, the major oncogenic mutations in LUAD in smokers were *EGFR* (40.4%), followed by *KRAS* (14.0%) and *ALK* (3.4%). In contrast, LUAD in non-smokers mostly had mutations in *EGFR* (71.6%), *ALK* (7.3%), and *FGFR* (4.7%) (16). Finally, telomere length is shorter in LUAD in smokers than in LUAD in non-smokers (17). A previous study using cytometry by time-of-flight revealed enrichment of follicular helper T (Th), Th0, Th1, γδT, natural killer (NK)/T, and effector CD8^+^ T cells in the TME of LUAD in smokers, suggesting that smoking may promote the immune response (18). However, differences in the TME of lung cancer according to smoking status are understudied.

In this study, we investigated the TME of LUAD and analyzed TME-enriched innate immune cells in smokers compared to non-smokers. We focused on plasmacytoid dendritic cells (pDCs), specifically enriched in LUAD of smokers, and their surface marker, toll-like receptor 9 (TLR9), and explored their possible relationship with ICI treatment responses.

## 2 Materials and methods

### 2.1 Study samples and ethical approval of the study

Samples were obtained from patients admitted to Gangnam Severance hospital, one of the affiliated hospitals of Yonsei University College of Medicine, for curative resection of LUAD between 2019 and 2022. This study was approved by the Institutional Review Board of our institution (IRB No. 3-2019-0299).

The inclusion criteria were as follows: 1) patients with surgically resectable stage lung cancer, 2) patients without distant metastasis as assessed by additional staging tests, including PET-CT and brain MRI, 3) patients with no prior history or treatment of cancer, 4) patients with a smoking history of 30 packs or more per year who had been smoking until the time of lung cancer diagnosis, 5) the period from smoking cessation to lung resection was less than one month, 6) patients who consented to provide residual Tu and adjacent NL samples, 7) the obtained lung nodule was pathologically confirmed as LUAD. Patients who received adjuvant or neoadjuvant chemotherapy were excluded.

### 2.2 Single-cell RNA sequencing, quality control, and data analysis

The process from sample preparation to the generation of scRNA sequencing data and cell type annotation used in this study has been described previously (19). Briefly, specimens obtained by surgical resection were transferred to the pathology laboratory and processed by a pathologist (YJ Cha). After confirmation of adenocarcinoma, the tissues were sent to Macrogen Korea^®^ (Seoul, Korea), where they were processed via the company’s standardized dissociation and sequencing processes. Low-quality data were removed using the DoubletFinder (v.2.0.3) R package and according to the percentage of mitochondrial genes (>10%) and gene counts (<200) in the Seurat (v.4.2.1) package. After integrating all datasets, the FindNeighbors and FindClusters functions were used to cluster cells into the major cell types. The barcodes of the epithelial cells from Tu with perturbation of chromosomal gene expression were secured through the InferCNV package v.1.12.0; these cells were defined as lung cancer cells (20, 21). Marker genes of the cell types were obtained using Seurat’s FindAllMarkers and FindMarkers functions. The marker gene sets used for the annotation of each cluster have been reported previously (**Online Supplemental Figure 1**) (19).

### 2.3 Tissue microarray analysis and animal study

TMA blocks comprising 372 LUAD tissue samples were prepared from archived paraffin-embedded tissue from patients who underwent upfront surgery for curative purposes. The basic characteristics of the TMA cases are listed in **Online Supplemental Table 1**. Kras^LSL-G12D^ mouse lung cancer tissues treated with ionizing radiation or cisplatin were obtained from previous studies and the induction and treatment of lung cancer was shown in **Online Supplemental Figure 2** (22, 23).

### 2.4 Immunohistochemical and immunofluorescence staining analyses

IHC and IF stainings were performed using the antibodies and conditions listed in **Online Supplemental Table 2**. Digital images were obtained from the stained slides to further analysis, using confocal microscope (ZEISS LSM 980, ZEISS, Oberkochen, Germany) and slide scanner (ZEISS Axioscan 7, ZEISS).

### 2.5 Calculation of TMB based on TCGA-LUAD data

Analysis was performed using Mutation Annotation Format (MAF) files and RNA-sequencing data with clinical, exposure, and family history files of TCGA-LUAD from the GDC database (https://portal.gdc.cancer.gov/). The TMB for each case was calculated using the tmb function of the maftools R package (https://www.https//portal.gdc.cancer.gov/bioconductor.org/packages/devel/bioc/vignettes/maftools/inst/doc/maftools.html).

### 2.6 Statistical analysis

Differentially expressed genes between the two clusters of interest were determined using the Wilcoxon rank-sum test, which is the default option in Seurat v.3.2.2, and adjusted *P*-values were obtained using Bonferroni correction. Differences in distribution between NL and Tu in the cluster of interest were determined by dividing the number of cells belonging to individual subclusters by the total number of cells belonging to the subcluster of the corresponding case and were compared using the unpaired Wilcoxon rank-sum test.

## 3 Results

### 3.1 Overview of the study design

**Figure 1:**
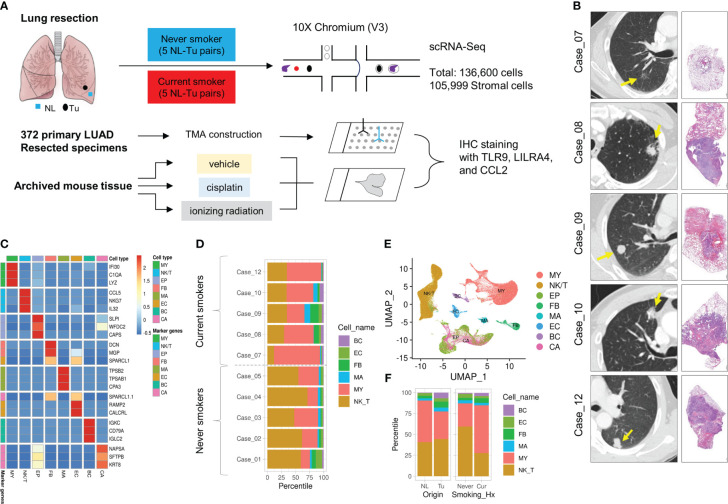
Overview of study design, cases included in this study and clustering of major cell populations. **(A)** Overview of this study. **(B)** High-resolution chest CT of tumors and representative microscopic images of tumors at low magnification (left lower bar = 50 μm). **(C)** Heatmap displaying the top expressed marker genes and major cell populations. **(D)** Horizontal stacked histogram showing the distribution of major cell populations in individual cases. **(E)** UMAP plot of the distribution of major cell populations. **(F)** Stacked histogram showing the distribution of major cell populations in terms of cell origin (left) and smoking history (right).

**Figure 2:**
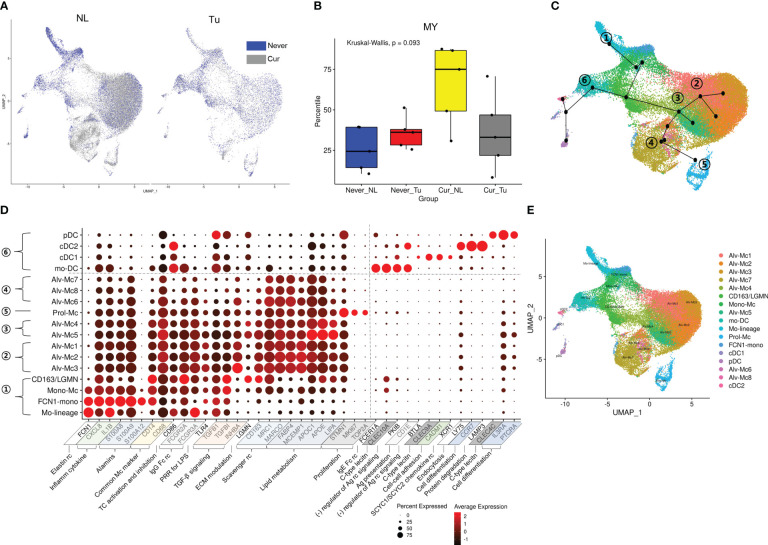
Distribution of MY cell population according to smoking history and tissue origin. **(A)** UMAP and **(B)** box plots depicting the proportional distribution of MY cells according to tissue origin and smoking history. *P-*value was obtained by Kruskal-Wallis test. **(C)** UMAP trajectories with Mo-linage cells as a starting point. **(D)** Dot plot of the genes used for annotation of the MY cell population. **(E)** UMAP with annotated MY cell populations.

Five pairs of non-tumor, normal-appearing lung tissues (NL), and LUAD tissues (Tu) were collected from current smokers. For comparison, data from five pairs of never-smokers were retrieved from our previous study (**Figures 1A, B**) (19). Current smoker patients underwent upfront surgery after at least two weeks of smoking cessation (**Table 1**). In total, 136,600 cells were obtained after quality control, and cancer cells were inferred using the InferCNV R package, as described previously (**Online Supplemental Figure 1**) (19). Each cluster was annotated using canonical biomarkers (**Figures 1C–E**). After excluding epithelial cells, the remaining 105,999 stromal cells were used for subsequent analyses. Among the major cell types, NK/T cells, mast cells, and myeloid (MY) cells showed different proportional distributions between NL and Tu, depending on smoking history (**Figure 1F**; **Online Supplemental Figure 3**). MY cells comprised the largest proportion of stromal cells in this dataset (46,704/105,599 cells, 44.2%) followed by NK/T cells (44,261 cells, 41.9%) and FB (4,530 cells, 4.3%) (**Figure 1D**; **Online Supplemental Table 3**). Especially in non-smokers, the MY cell level was higher in Tu to NL. However, in smokers, the MY cell levels were reversed, with lower levels in Tu and higher levels in NL (**Figures 2A, B**).

**Table 1:** Basic characteristics of the study cases.

	Case_07	Case_08	Case_09	Case_10	Case_12
Smoking (PY)	Current (50)	Current (40)	Current (47)	Current (40)	Current (40)
Stage (AJCC 8th Ed)	pT1bN0	pT3N2M0	pT1bN0	pT1bN0	pT1cN1
Size of tumor (cm)	1.8×1.7×1.5	2.3×2.3×1.8	1.8×1.5×1.5	1.8×1.8×1.5	2.2×2.0×1.5
Diagnosis	Invasive adenocarcinoma	Invasive adenocarcinoma	Invasive adenocarcinoma	Invasive adenocarcinoma	Invasive adenocarcinoma
Histologic component	Lepidic 60%, acinar 40%	Solid 95%, micropapillary 5%	Micropapillary 70%, acinar 30%	Acinar 90%, lepidic 10%	Acinar 50%, solid 40%, micropapillary 10%
EGFR mutation^*^	Wild type	Wild type	Wild type	L858R	Wild type

* PANAMutyper™ R EGFR mutation detection kit (real time PCR).

### 3.2 Smoking affects the distribution of the immune cells

Cigarette smoke contains particulates that are inhaled, and the lungs contain numerous immune cells, which serve as the first line of defense against inhaled particulates and respiratory pathogens (24). Our study aimed to investigate the impact of smoking on the distribution of major immune cell populations in NL and Tu.

Most of major cell clusters were distributed in higher proportions in Tu than in NL, except for the MY cell cluster, which was more enriched in NL than in Tu (**Figure 1F** left, **Online Supplemental Table 3**). When the distribution of major cell clusters was divided according to smoking status, NK/T constituted the largest fraction of cells in lung tissue from never smokers (59.6%), while MY was the most abundant in lung tissue from current smokers (57.3%) (**Figure 1F** right). In NL from smokers, MY accounted for the largest fraction, comprising 26,506 (71.9%) out of 36,857 cells obtained, which was more than twice the MY ratio (7,196 out of 21,961; 32.8%) in Tu from current smokers (**Online Supplemental Table 4**). But these differences did not reach statistical significance in the analysis according to tissue origin and smoking status; this is because the distribution of MY varied widely among individuals. Interestingly, there were no significant differences in the fraction of major cell clusters, except for BC, between non-smoker Tu and current smoker Tu. This also applied to MY, where they accounted for 33.5% in never smoker’s Tu and 32.8% in current smoker’s Tu, it accounted for 32.8%. In conclusion, smoking has a profound effect on the lung microenvironment. In Tu, where smoking particulates cannot be directly delivered into tissues, cellular compositional changes caused by smoking were smaller than that in NL.

### 3.3 Subclustering of MY cells

To in depth analyze the effects of smoking on MY, the MY cells were further divided into 17 subclusters: four subclusters related to tissue migration and differentiation, nine tissue-resident macrophage clusters, and four DC subclusters (**Figures 2C–E**; **Online Supplemental Table 5**; **Online Supplemental Figure 4A**). The subclusters involved in tissue migration and differentiation processes were as follows: 1) Mo-lineage: involved in the early stages of migration and differentiation. 2) FCN1-mono: characterized by overexpression of the elastin receptor FCN1. 3) Mono-Mc: characteristics of monocytes (IL1B and S100A8 expression) and macrophages (MRC1 and C1QB expression). 4) CD163-LGNM: characterized by overexpression of CD163 involved in clearance and endocytosis of hemoglobin/haptoglobin complexes in extracellular matrix modulation.

Differentiated tissue macrophages were divided into four subclusters according to the axis obtained from the slingshot simulation (25) as follows: 1) Alv-Mc 1, 2, and 3: having general characteristics of alveolar macrophages related to TGF-β signaling, scavenger receptor and apoptotic cell-uptake molecules, lipid handling and metabolism, and interaction with and breakdown of extracellular matrix components. 2) Prol-Mc: overexpressing proliferation markers, such as MKI67, STMN1, and TOP2A. 3) Alv-Mc 4, 5: remarkable overexpression of genes involved in lipid metabolism as well as alveolar macrophage markers. 4) Alv-Mc 6, 7, and 8: relatively low expression of inflammatory markers and TGF-β signaling.

Four DC subclusters were identified along an axis that developed in a different direction from macrophages. They could be clearly distinguished based on the expression of C-type lectin and activation markers of inflammatory cells (**Online Supplemental Figures 4B, C**) (26). 1) Monocyte-derived DCs (mo-DCs): characterized by overexpression of FCER1A, CLEC10A, and CD1C as they differentiate from monocytes during inflammation. 2) Conventional DC type I (cDC1): characterized by the expression of CLEC9A and CADM1 and effective antigen presentation to CD8^+^ T cells. 3) Conventional DC type II (cDC2): extensive expression of pattern recognition receptors and overexpression of LY75 and LAMP3 involved in endocytosis and protein degradation. 4) Plasmacytoid DCs (pDCs): characterized by nucleic acid uptake and degradation and secretion of type I interferon (IFN) along with overexpression of CLEC4C.

### 3.4 Cell composition of MY clusters differs according to tissue origin and smoking status

**Figure 3:**
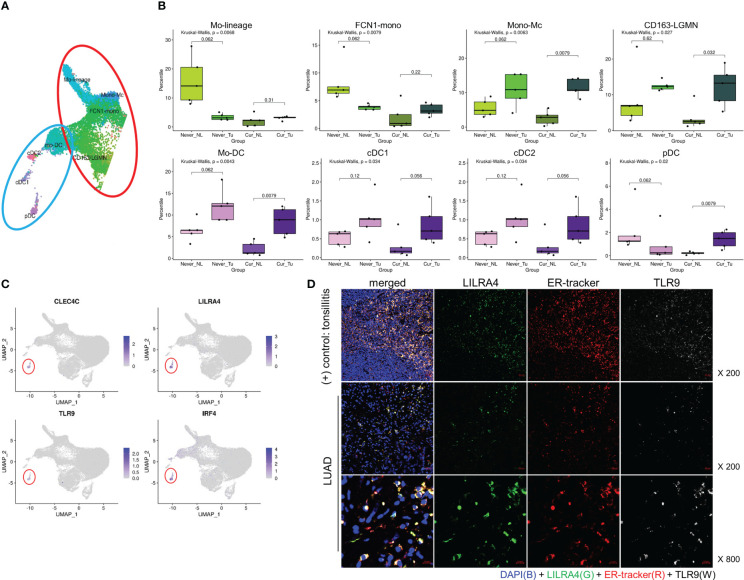
Tissue distribution of monocyte and DC populations, and markers of pDC. **(A)** UMAP and **(B)** box plots showing the distribution of the monocyte (upper) and DC (lower) populations. *P-*value was obtained by Kruskal-Wallis test, and for the comparison of paired values of NL-Tu, Wilcoxon signed rank sum test with paired option was used. **(C)** UMAP with the locations of representative pDC marker genes. **(D)** Immunofluorescence staining of LILRA4, ER-Tracker, and TLR9 in tonsil (top) and lung (mid and bottom) tissues.

Then we investigated the composition of the MY subclusters differed according to smoking status (never vs. current) and tissue origin (Tu vs. NL). The MY and DC subclusters had distinct cellular composition. Alv-Mc1 and Alc-Mc2, which had typical characteristics of alveolar macrophage and occupied the largest fraction in MY, were significantly increased in the NL of smokers (**Online Supplemental Figures 5A–E**). Meanwhile, the ratios of Mono-Mc, CD163-LGMN, Mo-DC, cDC1, and cDC2 were increased in Tu compared with that in NL, regardless of smoking status. Interestingly, the ratios of Mo-lineage, FCN1-mono, and pDCs were decreased in Tu compared with that in NL in non-smokers, suggesting that smoking and intratumoral factor(s) might have a compound effect on the tissue distribution of these cell populations (**Figures 3A, B**). Taken together, the tissue distribution pattern of MY could be classified into 3 groups; (1) typical alveolar macrophages (Alv-Mc1 and Alc-Mc2) which were enriched in smokers’ NL where highly affected by smoking, (2) Mono-Mc, CD163-LGMN, Mo-DC, cDC1, and cDC2 which recruited into Tu regardless smoking status, and (3) Mo-lineage, FCN1-mono, and pDCs, which distribution was influenced by compound effect of smoking and intratumoral factor(s). Additional analysis was performed by selecting pDCs that were terminally differentiated and had a significant difference in distribution between NL and Tu of smokers.

### 3.5 Enrichment of pDC in current smokers’ Tu is associated with a favorable prognosis

**Figure 4:**
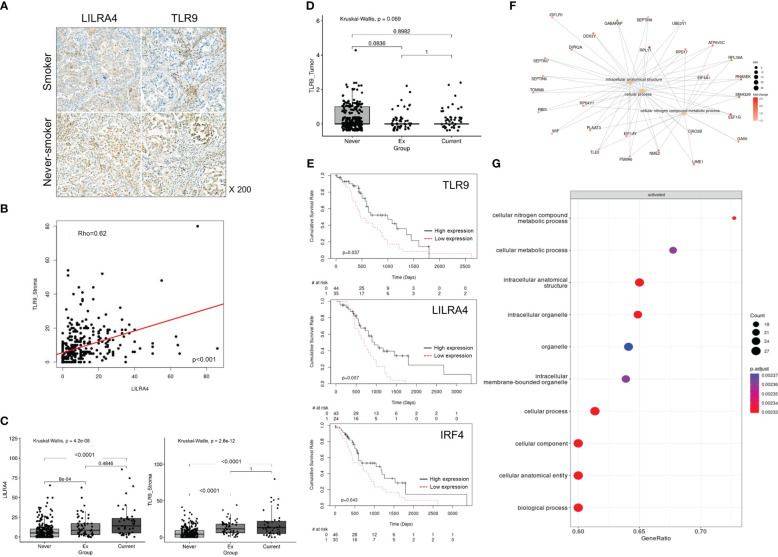
Characteristics of pDCs in the TME in smokers and clinical implication of their enrichment. **(A)** Representative IHC staining of LILRA4 and TLR9 in a TMA comprising 372 LUAD tissue samples. **(B)** Correlation between LILRA4 and TLR9 expression in stromal immune cells in LUAD. *P-*value was obtained by Spearman’ correlation test. **(C)** Box plot comparing TLR9 and LILRA4 expression in stromal immune cells in LUAD according to smoking history. **(D)** Box plot comparing TLR9 expression in tumor cells of LUAD according to smoking history. In **(C)** and **(D)** *P-*value was obtained by Kruskal-Wallis test and *post hoc* analysis was performed using Bonferroni correction. **(E)** Survival analysis according to the expression of TLR9 (top), LILRA4 (mid), and IRF4 (bottom) in TCGA-LUAD cases (adjusted for age, sex, and smoking history). *P-*value was obtained by Cox proportional hazard model. **(F)** Caterpillar and **(G)** dot plots showing the characteristics of pDCs in Tu of smokers compared with those of non-smokers.

In order to confirm whether pDC is enriched in smoker Tu, we first tried to find biomarkers that could complement its morphological feature. In scRNA dataset, pDCs were distinguished from other MY subpopulations by the overexpression of *CLEC4C, LILRA4, TLR9*, and *IRF4* (**Figure 3C**). *LILRA4* and *TLR9* were selected based on their well-delineated expression in multiplex fluorescence staining, using non-tumor tonsil tissue as a control (**Figure 3D**). In Tu, approximately 70% of LILRA4-positive cells co-expressed TLR9 (data not shown). To investigate the spatial distribution of pDCs, IHC staining of LILRA4 and TLR9 was performed using TMA blocks composed of 372 LUAD, and their expression in stromal immune cells and cancer cells was compared (**Figures 4A–C**; **Online Supplemental Figure 6**). In immune cells, there was a significant positive correlation between the expression of LILRA4 and TLR9. The expression levels of LILRA4 and TLR9 in immune cells were higher in individuals with a smoking history compared to those without a smoking history. However, in cancer cells, the expression of TLR9 was not significantly associated with a history of smoking (**Figures 4C, D**). To find out the clinical implication of pDCs’ enrichment in Tu, the pDC-related markers in LUAD were evaluated using the open-source datasets TCGA-LUAD and OncoLnc (http://www.oncolnc.org/) (**Figure 4E**; **Online Supplemental Figure 7**). Overexpression of the pDC-related markers, TLR9, LILRA4, and IRF4, was associated with superior overall survival when comparing the top 25% overexpressing patients with the remaining patients. In summary, LILRA4 and TLR9 were useful as pDC markers, and they were overexpressed in the immune cells of Tu obtained from patients with a history of smoking. In addition, overexpression of pDC-related genes was associated with favorable clinical outcomes.

### 3.6 CCL2 and TMB might related to the recruitment of pDCs to smoker’s Tu

**Figure 5:**
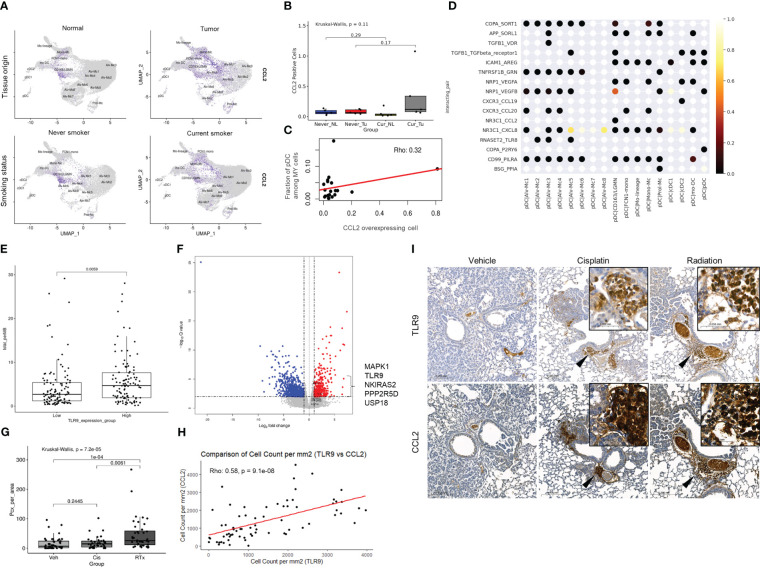
Environmental factors involved in pDC migration in smokers’ Tu and the relationship between TLR9 overexpression and anti-cancer treatment. **(A)** CCL2 plotted on a UMAP plot of the MY cell population according to tissue origin and smoking history. **(B)** Boxplot showing the fraction of CCL2 (+) cells according to the smoking and tissue origin. **(C)** Correlation plot showing the relationship between CCL2 overexpressing cell count and pDC fraction among MY cells. **(D)** Visualization of the interactions between pDCs and the remaining MY cell populations using CellPhoneDB. **(E)** A boxplot comparing TMB between the top 25% of TLR9 mRNA expression group vs. bottom 25% group in the TCGA-LUAD dataset. *P-*value was obtained by Welch’s two-sample t-test. **(F)** A volcano plot showing differentially expressed genes in the high TMB group (≥20 mutations/Mb) compared with that in the low TMB group (<5 mutations/Mb) in the TCGA-LUAD dataset. Among the genes belonging to the TLR9 cascade of Reactome (https://reactome.org/), those with log_2_FC ≥ 1 and adj*P* ≤ 0.05 were marked in the plot. **(G)** Differential TLR9-expressing immune cell infiltration in the treatment groups. *P-*value was obtained by Kruskal-Wallis test and *post hoc* analysis was performed using Bonferroni correction. **(H)** Correlation of TLR9 and CCL2 expression in mouse model. **(I)** TLR9 and CCL2 IHC stainings in Kras^LSL-G12D^ mice after treatment with cisplatin or ionizing radiation. Inlets show higher magnification view of area indicated by arrowheads.

Previously, it was suggested that the distribution of pDCs might be influenced by compound effect of smoking and intratumoral factors(s). We divided the factors that shifted the pDC to smoker’s Tu into the effects of smoking and the intratumoral factor(s), and then investigated the smoking-related factor first. In the TME of smokers, pDCs showed enrichment of pathways related to intracellular anatomical structures and cellular nitrogen compound metabolism (**Figures 4F, G**; **Online Supplemental Table 6**). Among the factors reported to be associated with pDC mobilization, *CCL2, CMKLR1, CCR6, C3AR1, C5AR1, ADORA1*, and *CXCR4* (27), the overexpression of *CCL2, CXCR4, C3AR1*, and *CMKLR1* were observed in MY cells from Tu of smokers (**Online Supplemental Table 7**). Uniform manifold approximation and projection (UMAP) plots revealed increased expression of CCL2 in the MY subsets, FCN1-mono and CD163-LGMN, of Tu in smokers (**Figure 5A**; **Online Supplemental Table 5**). Observing the localization of CCL2 overexpressing cells from another point of view, they were enriched in the current smokers’ Tu, and the fraction of CCL2 overexpressing cells and pDC tended to show a positive correlation (**Figures 5B, C**). When inferring molecules that interact with CCL2 using CellPhoneDB, a significant interaction between CCL2 and NR3C1 was estimated (**Figure 5D**). NR3C1 was overexpressed in pDCs (**Online Supplemental Table 5**), and it is involved in inflammatory responses, cellular proliferation, and differentiation in target tissues, which is worthy of further study (28).

Then intratumoral factor(s) that forced to move pDC into smoker’s Tu was investigated. Clinical, pathological, and laboratory parameters related to TLR9 overexpression were retrieved from a TCGA dataset. The TMB was significantly higher in the TLR9 overexpression group (5.81 mutations/Mb vs. 4.36/mutations Mb, *P* = 0.0059, Welch’s two-sample *t*-test, **Figure 5E**). The TCGA-LUAD dataset was divided into three groups based on TMB: ≤5 mutations/Mb, low TMB; 5–20 mutations/Mb, intermediate TMB; and ≥20 mutations/Mb, high TMB. The high TMB group showed a significant increase in TLR9 expression (1.5214 log_2_FC) compared with that in the low TMB group (adj*P* = 0.0050) (**Figure 5F**; **Online Supplemental Table 8**), suggesting a possible link between TLR9 expression and TMB. Taken together, the recruitment of pDC to smokers’ Tu might be influenced by complex factors of TMB and CCL2, which was overexpressed in FCN1-mono and CD163-LGMN in smokers’ Tu.

### 3.7 DNA damaging anticancer treatment might recruit pDC

pDC is known to sense bacterial unmethylated CpG-rich DNA through TLR9 and mediate the subsequent inflammatory response, but reports on mammalian DNA sensing are limited. To investigate whether pDCs play an additional role in sensing damaged mammalian DNA related to therapy, the KRAS^G12D^-driven mouse lung cancer model (Kras^LSL-G12D^ mice) was used to evaluate the effect of radiation on TLR9 expression.

**Figure 6:**
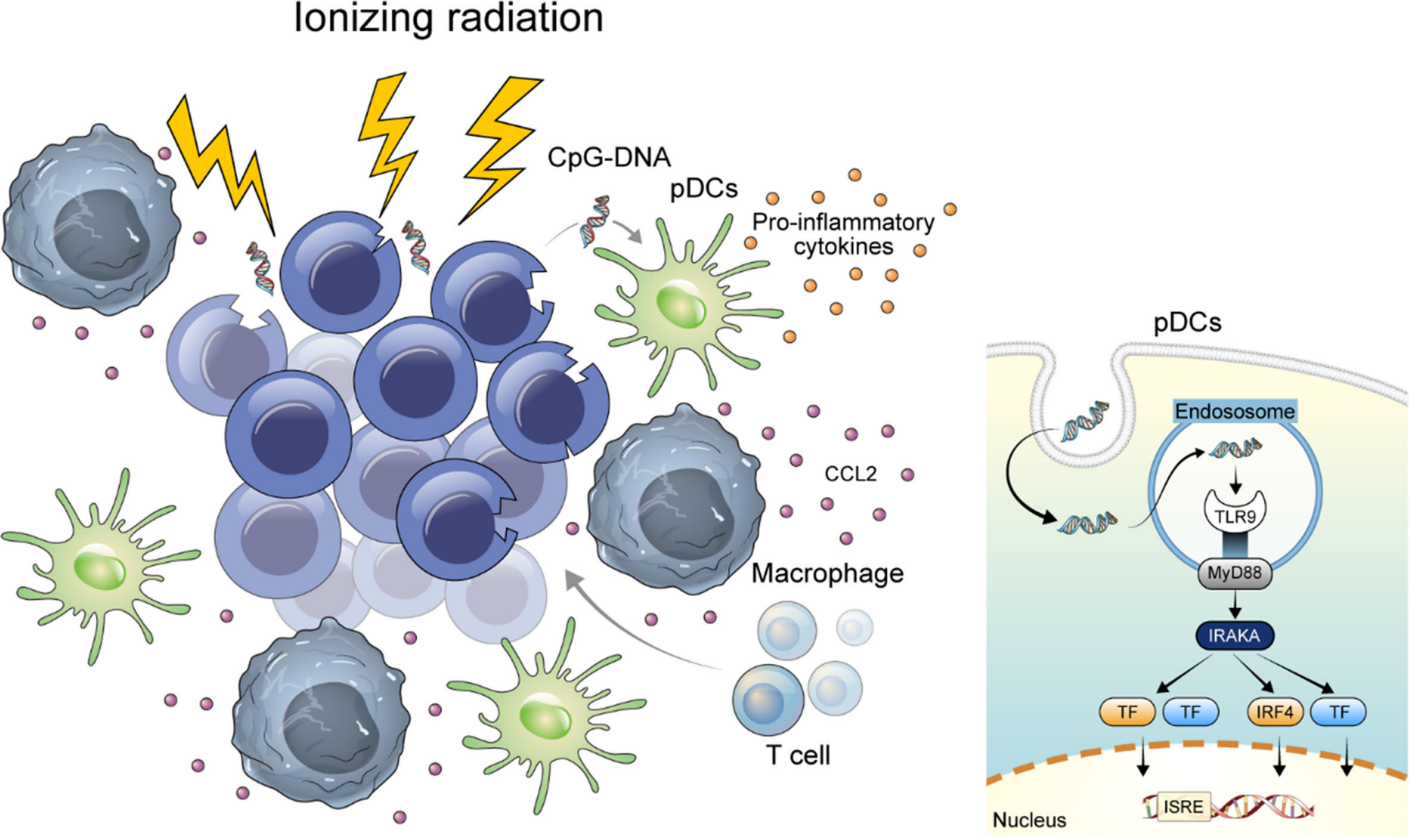
Graphical abstract of this study. DNA released from damaged mammalian cells may positively regulate TLR9 expression through CCL2.

Expression of TLR9 and CCL2 in the lungs of mice either treated with 5 mg/kg (ip) of cisplatin per week 2 times or treated with 10 Gy of ionizing radiation once were compared with those from mice treated with vehicle. Lungs were harvested in 2 weeks from the start point of treatment (**Online Supplemental Figure 2**). TLR9-expressing immune cells were mainly accumulated in the peribronchial capillaries near the tumor, whereas normal mouse lung of same strain and vehicle-treated tissues lacked TLR9-expressing immune cells. Although accumulation of TLR9-expressing immune cells was observed around the tumor in both the cisplatin-treated and the ionizing radiation-treated group, TLR9-expressing immune cells were significantly increased in the lung of ionizing radiation-treated group compared with that in the vehicle treated group (**Figures 5G–I**). In addition, the number of TLR9-expressing immune cells in mouse lung tissue showed a significant correlation with the number of CCL2 expressing cells (**Figure 5H**). Collectively, these findings support the hypothesis that DNA released from damaged mammalian cells may positively regulate TLR9 expression through CCL2 (**Figure 6**).

## 4 Discussion

To discover biomarkers for the response to ICI treatment, we dissected the biological space into quadrants, smokers vs. non-smokers and Tu vs. NL, and then investigated cell clusters specifically enriched in the Tu of smokers.

In our scRNA dataset, pDCs were one of the MY subclusters and were significantly enriched in the Tu of smokers. TCGA-LUAD RNA-sequencing data were analyzed to validate that the pDC signature was specifically overexpressed in smokers. In smokers, *CLEC4C, LILRA4*, and *TLR9* levels were consistently higher than those in non-smokers; however, statistical significance was not reached, probably because of the immense proportion of epithelial components, including cancer cells (data not shown).

Several studies have investigated the association between pDCs and TLR9 expression in lung cancer (29–35). pDCs are a subset of innate immune cells that recognize viral or bacterial nucleic acids and secrete type I interferon (IFN) (27, 36). pDCs secrete proinflammatory cytokines, such as IFN, IL6, IL12, CXCL8, CXCL10, CCL3, and CCL4, leading to an immune-prone TME (27).

pDCs have been previously defined as CD4^+^/IL3RA(CD123)^+^/CLEC4C(BDCA2)^+^ cells (33). In the present study, the pDC subset was distinguished by the expression of *CLECL4, LILRA4, TLR9*, and *IRF4*. CLEC4C binds to non-sialylated galactose-terminated biantennary glycans containing the trisaccharide epitope Gal(beta1-3/4)GlcNAc(beta1-2)Man (37, 38) and aids in the presentation of ligands to T cells (39). LILRA4 is a coreceptor that controls innate immune responses generated through FCER1G in viral infection (40, 41) and acts as a negative regulator of TLR7 and TLR9 signaling cascades (40–42). TLR9 and TLR7 are representative pattern recognition receptors that recognize single-stranded RNA and unmethylated CpG DNA, respectively (27). IRF4 is a transcriptional regulator of type I IFN-dependent immune responses and plays a critical role in the innate immune response against DNA and RNA viruses (43). From the function of pDCs’ marker genes, the relationship between pDCs and damaged DNA could be inferred and they may be used as treatment response indicators for DNA damaging anti-cancer treatment. Among the pDCs’ marker genes, *CLEC4C* could not be evaluated for its prognostic value, not because it was not associated with prognosis, but because it was not listed in OncoLnc’s gene set.

pDCs in peripheral blood were significantly increased in patients with advanced-stage NSCLC and smoking history (33), which is in line with our finding that pDCs were enriched in the TME in smokers with LUAD. In addition, pDCs interfered with the antitumor effect of CpG-oligodeoxynucleotides in a syngeneic mouse lung cancer model established using Lewis lung carcinoma cells (35). Depletion of pDCs resulted in a decrease in lung tumor burden accompanied by enhanced innate immunity as indicated by an influx of active myeloid DCs and CD8^+^ T cells with strong production of Th1- and Th17-like cytokines (35). In contrast to the findings in the mouse model, we found that the pDC signature was associated with a favorable prognosis in the TCGA-LUAD database. This may be attributed to intrinsic differences in tumors or host species, as the previous study used a syngeneic Lewis lung carcinoma model developed from a mouse epidermoid carcinoma, not from a human adenocarcinoma. Alternatively, it may be possible that artificial depletion of pDCs in experimental animal models potentiated the anti-cancer immune response through the activation of other damaged DNA-sensing pathways, i.e. AIM2 or cGAS-STING pathways, with DNA remaining in the TME.

Belmont et al. reported that TLR9 expression in tumor-infiltrating immune cells was associated with worse survival in early-stage NSCLC patients (31). They also studied *TLR9*-knockout K-ras^LA1^ lung cancer mice (K-ras^LA1^TLR9^−/−^), and observed shorter survival in K-ras^LA1^TLR9^+/+^ mice (31). However, the authors may have overlooked the fact that TLR9 is specifically expressed in pDCs, not in all inflammatory cells. We speculated that TLR9 knockout in mice could potentially improve prognosis by suppressing pDCs, although further validation should be performed. In other study using fluorescence-assisted cell sorting to analyze human NSLCL tissues, intratumoral pDCs had an immunosuppressive phenotype, determined by the expression of CD33 (SIGLEC3) and CD274 (PD-L1) (34). Meanwhile, Perrot et al. reported that tumor-associated pDCs seem to be in an immature state as they lack CD80/B7-1, CD86/B7-2, CD83, and LAMP/CD208 (32). Despite their immaturity, tumor-associated pDCs appear to have an intrinsic ability to migrate to draining lymph nodes as antigen-presenting cells (32). These heterogeneous reports indicate that the role of pDCs in lung cancer TME has yet to be elucidated. In addition, further studies are required to determine whether pDCs themselves or in which conditions of pDCs are suitable targets for treatment.

The positive correlation between TLR9 expression and the TMB in lung cancer suggested that pDCs may act as a surrogate marker for a high TMB in lung cancer. We also observed pDC accumulation in peribronchial capillaries of Kras^LSL-G12D^ mice treated with ionizing radiation or a DNA-alkylating agent. As pDCs detect pathogen-derived nucleic acids, which could be tumoral DNA debris, accumulation of pDCs in the TME might be enabled by anti-cancer treatments, such as chemotherapy and/or radiation therapy. However, the activation of pDCs is far more critical, considering that most pDCs are in a hypofunctional state, which was also consistent with what we observed in the our scRNA dataset (44, 45).

This study had several limitations. First, we focused on the TLR9 pathway among the three damaged DNA-sensing pathways of pDCs, i.e., the TLR9, AIM2, and cGAS-STING pathways. TLR9 is the major pathway in the recognition of damaged extracellular DNA in pDCs, whereas the AIM2 and cGAS-STING pathways are mainly involved in the removal of intracellular DNA. Additional studies are required to determine the involvement of the other two pathways. Second, the mechanism of TLR9 overexpression in tumoral pDCs of smokers could not be explored. Reports on TLR9 overexpression induced by damaged mammalian DNA are limited. An et al. reported that lipopolysaccharide stimulation upregulated TLR9 expression via the NF-κB, ERK, and p38 MAPK signaling pathways, which seems irrelevant to our study as they analyzed TLR9 expression in macrophages (46). Finally, whether pDCs directly mediate ICI treatment could not be determined. As several clinical studies on the combination of ICI and TLR9 agonists and pDC activators are underway, further elucidation of the role of pDCs is expected. We expect that our study provided valuable background data for further clinical studies.

In conclusion, our study indicates that cancer cell-derived DNA can evoke an immunogenic reaction along with the recruitment of pDCs. This may boost ICI treatment effects in lung cancer patients, regardless of smoking status. Further investigation of the molecular basis of the TME in lung cancer and endeavor to engineer the innate immune compartment may aid in developing more effective ICI treatment in NSLCL patients.

## Data availability statement

The datasets presented in this study can be found in online repositories. The names of the repository and accession number(s) can be found below: https://www.ncbi.nlm.nih.gov/sra, PRJNA901260.

## Ethics statement

The studies involving human participants were reviewed and approved by Institutional Review Board of Gangnam Severance Hospital (IRB No. 3-2019-0299). The patients/participants provided their written informed consent to participate in this study.

## Author contributions

YJCha Collection and processing of specimens, Data curation, Formal analysis, Investigation, Resources, Visualization, Writing – original draft, Writing – review and editing. EYK Data curation, Formal analysis, Investigation, Software, Validation, Visualization, Writing – original draft, Writing – review and editing. YJChoi IRB work and securing informed consent, Clinical data curation, Formal analysis. CYK Data curation, Formal analysis, Software, Validation, Visualization. MKP Experimental investigation. YSC Conceptualization, Recruitment of study subjects, Data curation, Formal analysis, Funding acquisition, Investigation, Project administration, Resources, Software, Supervision, Validation, Visualization, Writing – original draft, Writing – review and editing. All authors contributed to the article and approved the submitted version.
