# Supplementary figures and images for: Accumulation of plasmacytoid dendritic cell is associated with a treatment response to DNA-damaging treatment and favorable prognosis in lung adenocarcinoma
Source: Front Immunol. 2023 Jun 26;14:1154881. doi: 10.3389/fimmu.2023.1154881 (PMC10330699; doi:10.3389/fimmu.2023.1154881)

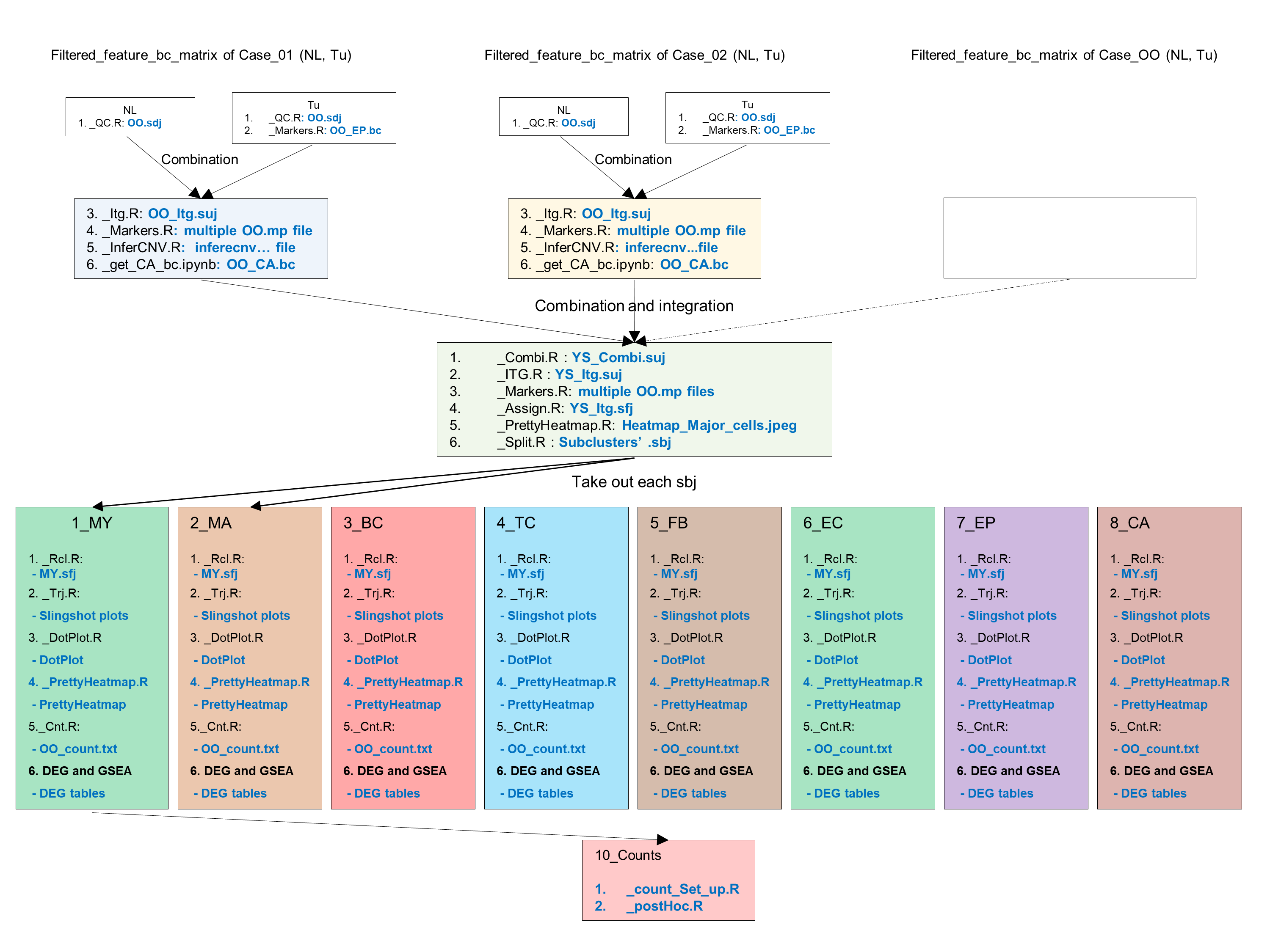

Supplement: Supplementary file 1 [file Image_1.tif]

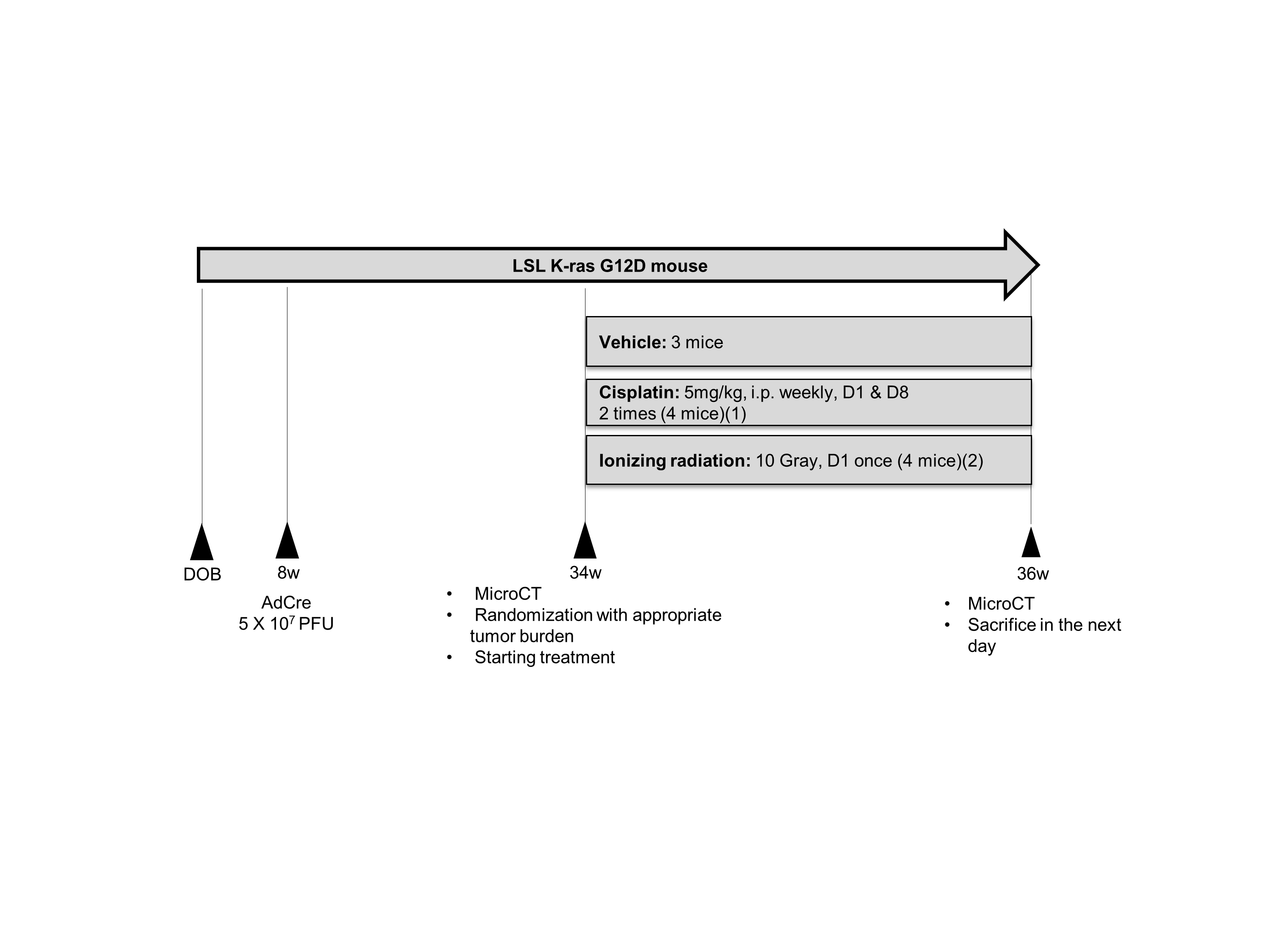

Supplement: Supplementary file 2 [file Image_2.tif]

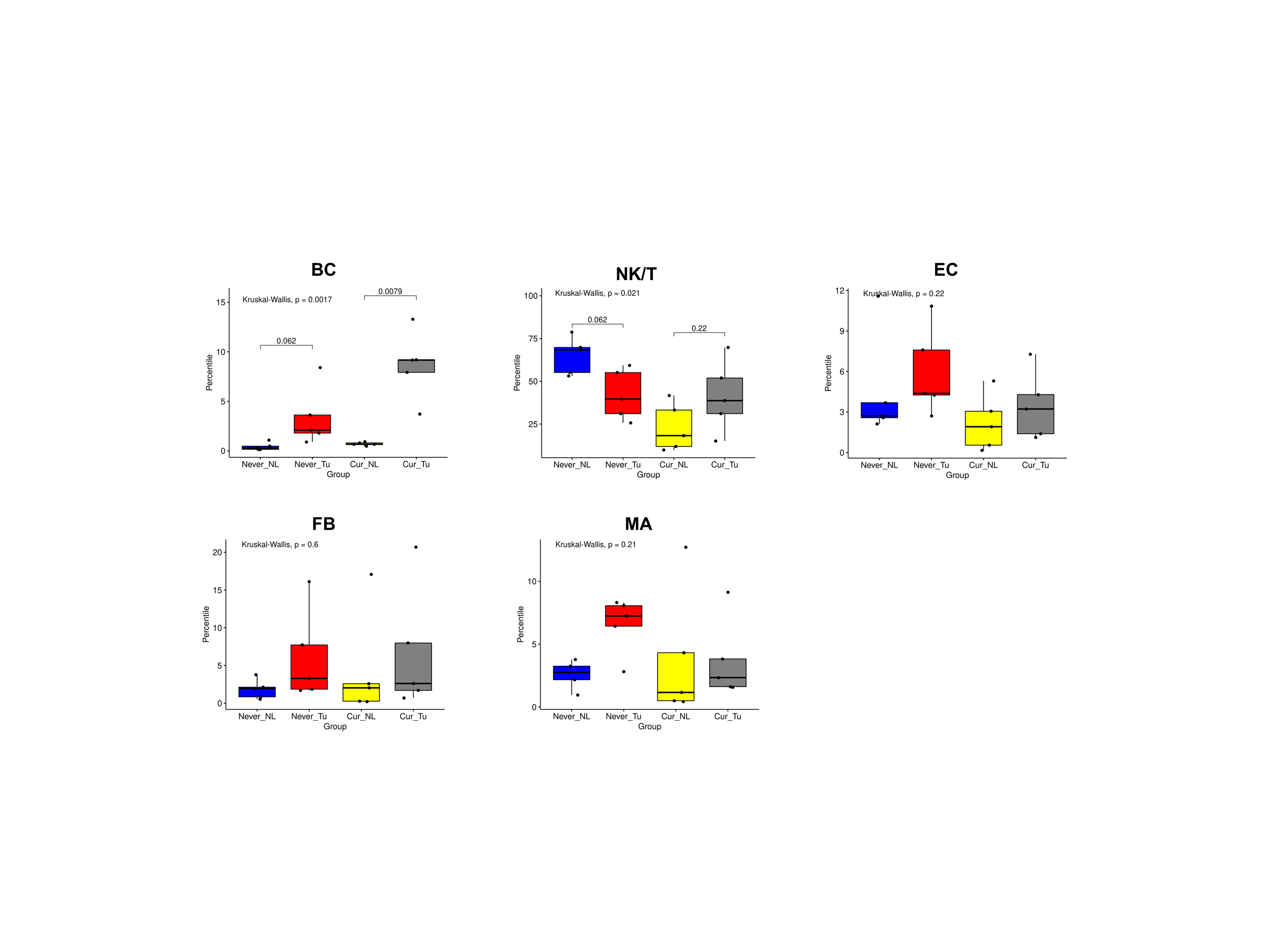

Supplement: Supplementary file 3 [file Image_3.tif]

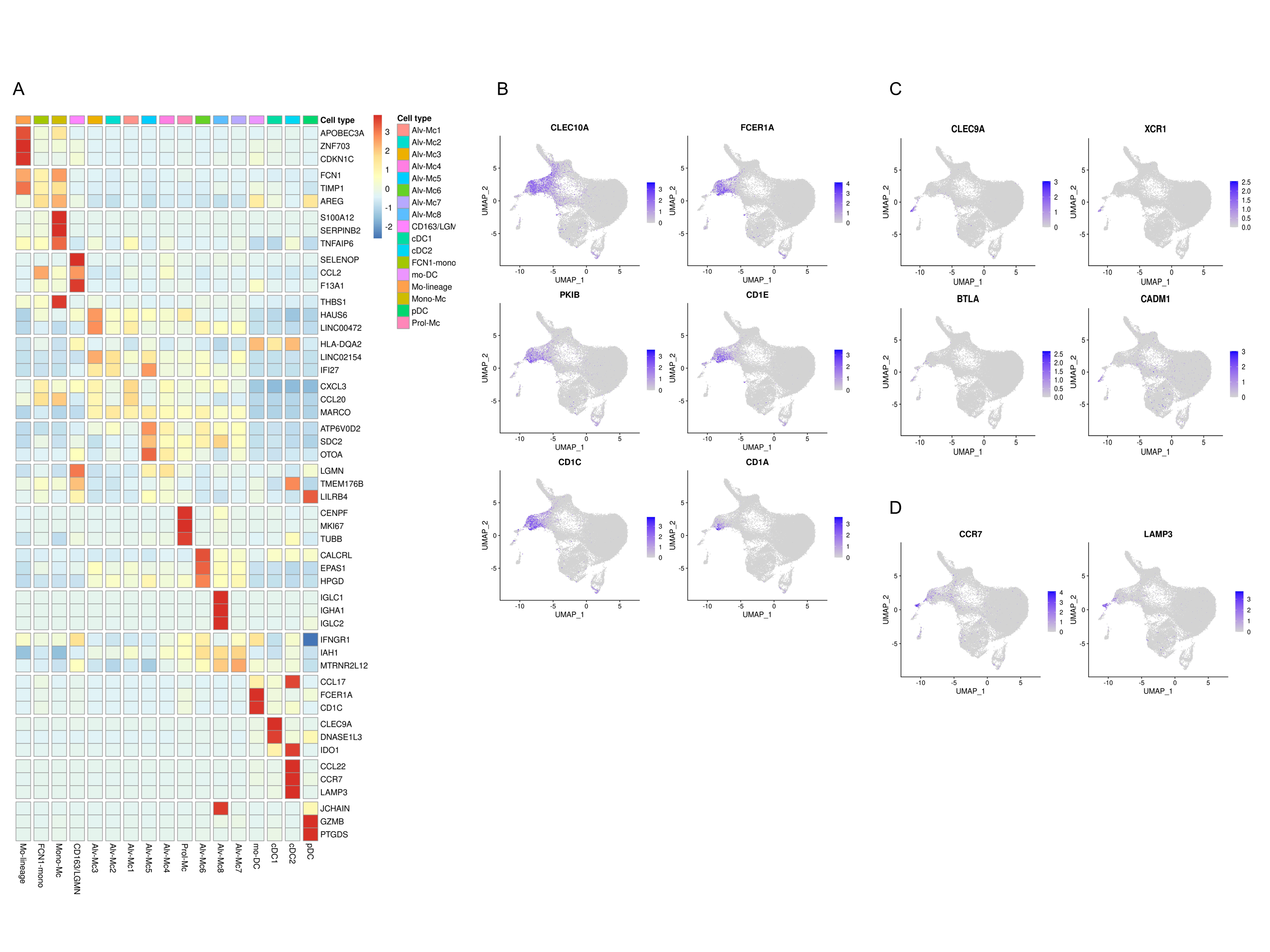

Supplement: Supplementary file 4 [file Image_4.tif]

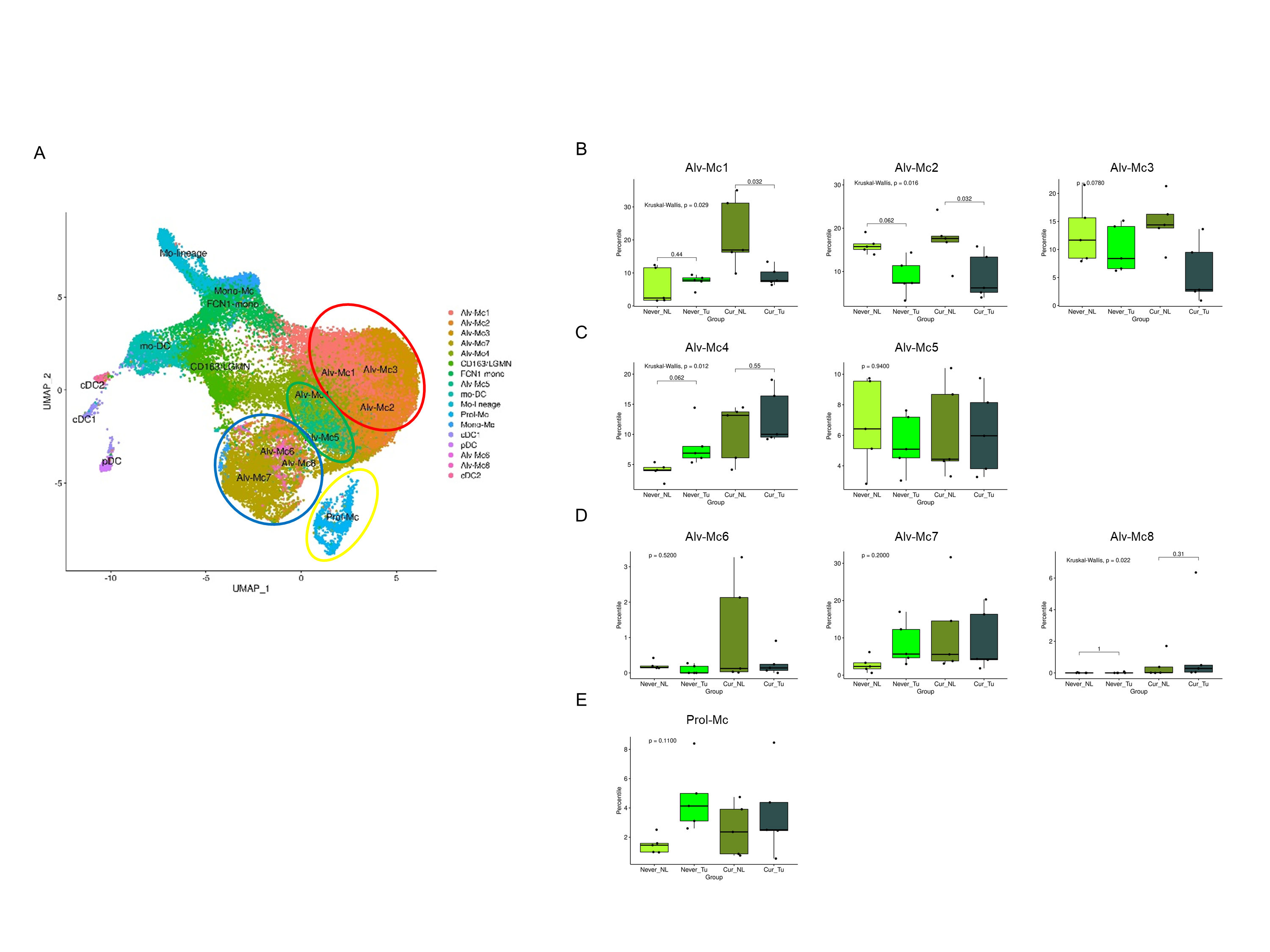

Supplement: Supplementary file 5 [file Image_5.tif]

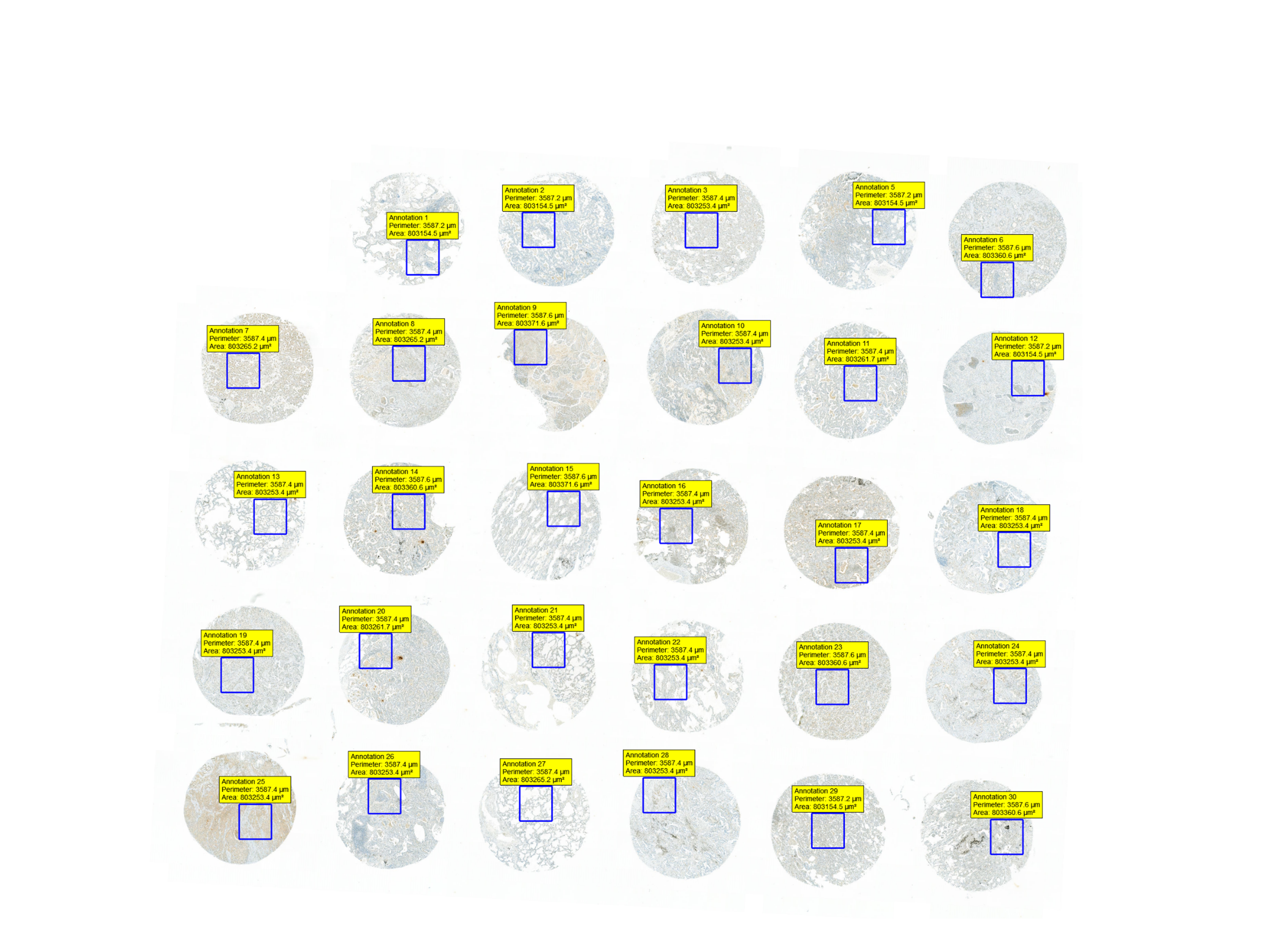

Supplement: Supplementary file 6 [file Image_6.tif]

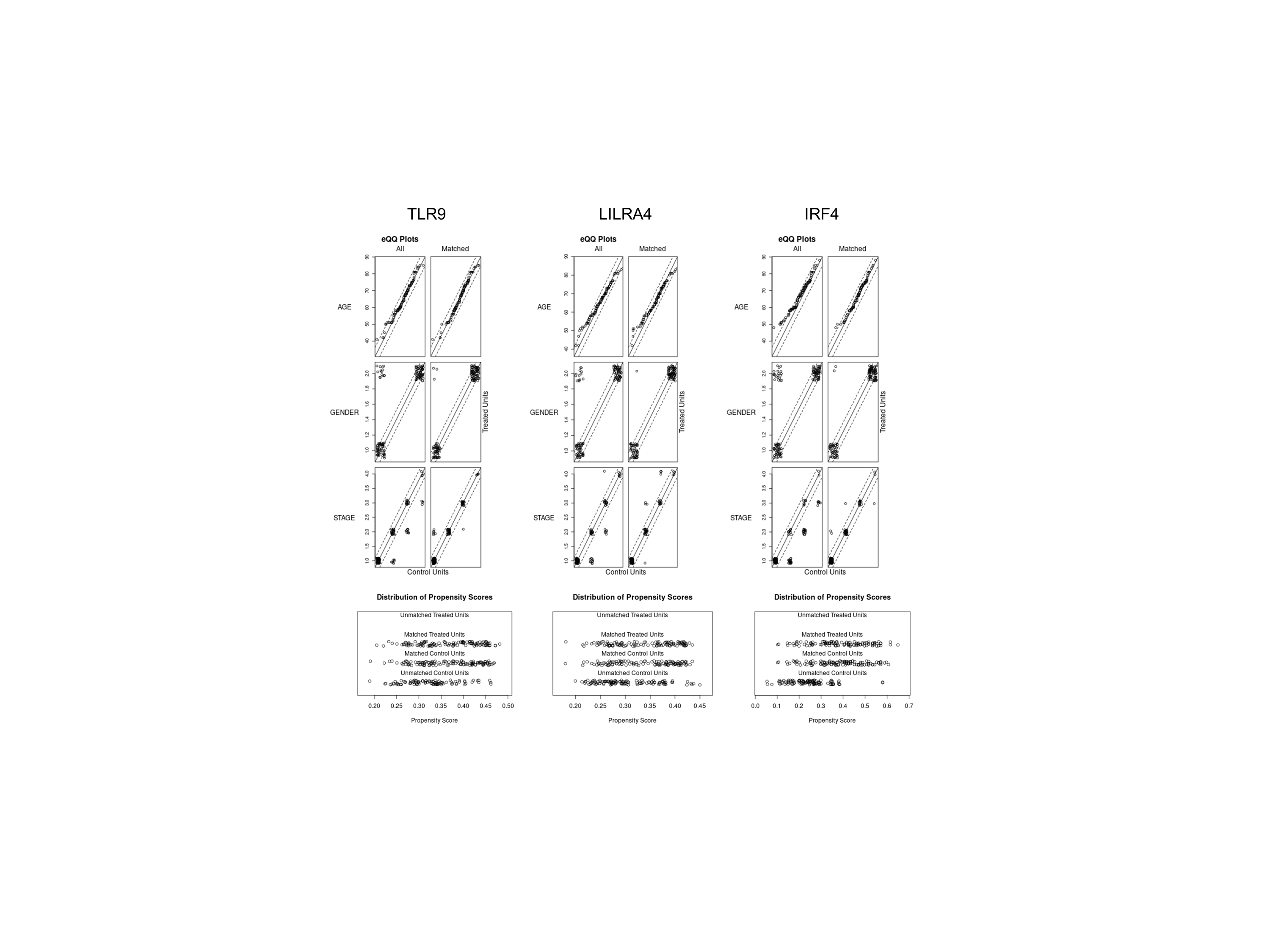

Supplement: Supplementary file 7 [file Image_7.tif]
